# The Importance of Feedstock and Process Control on the Composition of Recovered Carbon Black

**DOI:** 10.3390/polym17212913

**Published:** 2025-10-31

**Authors:** Christopher Norris, Antonio Lopez-Cerdan, Peter Eaton, Richard Moon, Mark Murfitt

**Affiliations:** 1 Murfitts Industries, Station Road, Lakenheath, Suffolk IP27 9AD, UK; antonio.lopez-cerdan@murfittsindustries.com (A.L.-C.); mark@murfittsindustries.com (M.M.); 2 The Bridge, Joseph Ruston Building, University of Lincoln, Edgewest Road, Lincoln, Lincolnshire LN6 7EL, UK; peaton@lincoln.ac.uk; 3 Materials Innovation, Avon Protection, Hampton Park West, Semington Road, Melksham, Wiltshire SN12 6NB, UK; richard.moon@avon-protection.com

**Keywords:** rCB, pyrolysis, waste tyres, recycling, rubber, reinforcement, sustainability

## Abstract

Pyrolysis has emerged as a commercially viable material recovery process that supports circularity in the tyre industry. Here, it is demonstrated that a high degree of control can be imparted over the UK tyre waste stream and that statistically different feedstocks can be used to produce different grades of rCB based on their ash contents. The lower ash content rCB produced from truck tyres had superior in-rubber properties, closely matching those of the N550 reference. Silica, when not paired with a coupling agent, is known to be less reinforcing than CB, lowering the reinforcing behaviour of the high ash content rCB variant produced from car tyres. This justifiably places ash content within the classification and specification development discussion. However, a proximate analysis of UK waste tyres suggests that the typical rCB ash specifications of <20 wt% are unrealistic. Such limits would force producers to consider modifying process conditions to allow the deposition of carbonaceous residues to artificially dilute the ash content. This study investigates this process philosophy but conclusively demonstrates that carbonaceous residue is more detrimental to rCB performance than ash content. As such, carbonaceous residue content demands far more attention from the industry than it is currently afforded.

## 1. Introduction

The UK generates approximately 50 million waste tyres, equivalent to 700,000 tonnes, each year. Since the EU ban on landfilling came into effect in 2003 (EC Directive 1999/31), most of these waste tyres have been utilised in civil engineering applications, sports and recreational surfaces and for energy recovery as fuel in cement kilns [1]. The emergence of policy drivers to combat climate change and to promote material circularity, such as the European Commission Green Deal [2], has increased pressure on the industry to recover materials from waste tyres. Now, all the major tyre brands have imposed stringent sustainability goals, with most targeting the construction of tyres from 100% sustainable materials by 2050. This necessitates a movement away from traditional mechanical recycling technologies to those that can chemically deconstruct waste tyres into reusable raw materials. Pyrolysis has emerged as the frontrunning commercially capable material recovery process to support circularity in the tyre industry. As well as offering recovered products with low global warming potential and avoiding the consumption of fossil resources [3,4], properly controlled tyre pyrolysis comes with minimal emissions or residual waste [5].

Pyrolysis is defined as the thermal decomposition of organic materials in the absence of oxygen [6]. In the case of tyres, this involves cracking the long organic polymer chains into a hot gas phase, which consists of low-molecular-weight non-condensable gas and condensable tyre pyrolysis oil (TPO) fractions. The removal of organic matter leaves behind a solid mixture consisting of carbon black (CB), inorganic compounding ingredients and other pyrolysis residues, defined as ‘raw recovered carbon black (rCB)’ by ASTM committee D36 [7]. Assuming the raw rCB is of sufficient quality, it undergoes further milling and pelletising processes to alter its physical properties to meet the specification requirements of the end user [8]. This paper focusses on demonstrating how the properties of rCB are controlled by both feedstock selection and pyrolysis conditions.

The key to any material recovery process, including pyrolysis, is to produce consistent products that always meet the specification requirements of the end user. This poses a significant challenge to rCB producers, given that tyres are structurally complex articles comprising several different rubber components, including the tread, carcass and sidewall, alongside steel and textile reinforcements [9]. Each of the rubber components will typically contain one or more elastomers, reinforcing fillers such as CB and silica, plasticising agents, antidegradants, processing aides, curatives and other additives. It has been reported that more than one hundred ingredients can be added to the tyre, the precise nature of which depends on its specific use requirements [10,11]. To tackle the challenges associated with product consistency, it seems prudent to narrow the compositional variance of pyrolysis feedstocks by segregating the waste stream into structurally and compositionally similar tyres. However, it is acknowledged that manufacturer variability will persist even when segregating the waste stream by type. In the UK and the EU, the two main classes of tyres are passenger car and truck, accounting for approximately 70 wt% and 20 wt% of the total tyre waste stream, respectively. Passenger car tyres contain a higher synthetic rubber content and a greater utilisation of silica filler and textile reinforcements [12]. In contrast, truck tyres contain higher levels of steel, natural rubber and carbon black [13]. An additional benefit of feedstock selection is to narrow the aggregate size distribution of the resultant rCB, as passenger car and truck tyres contain different grades of CB. A broader aggregate size distribution has been linked to a reduced reinforcing potential for rCB [8,14].

The first aim of this study was to assess the ability to produce compositionally different pyrolysis feedstocks by segregating car and truck tyres from the UK waste stream, and, further, to assess the impact on the composition and reinforcing ability of the resultant rCB products. The major difference between the feedstocks is the higher silica content of the passenger car tyre, arising from the drive to enhance fuel efficiency following the introduction of labelling rules by the European Commission in 2012 [15]. Assuming a complete pyrolysis of the tyre rubber, the resultant solid rCB product is a mixture of CB, silica and other inorganic compounding ingredients. rCB produced from passenger car tyres is, therefore, expected to contain a higher silica (ash) content. The ash content of an rCB has been reported as an influential factor on rubber reinforcing potential [16,17,18], the dominant factor being that silica, when not paired with a coupling agent, is a less reinforcing filler than CB, with laboratory produced rCB samples demonstrated to decrease in reinforcing potential with increasing silica content [8]. It is also documented that silica interacts with zinc during compounding, leading to the removal of soluble zinc from its normal accelerator activating function, subsequently reducing crosslink density [19]. Based on these observations, it is understandable that tyre manufacturers have developed rCB specifications that stipulate a requirement for ash content to be ≤20 wt% [20]. However, these observations are only relevant to ‘clean’, low-residue-content rCB produced from the complete pyrolysis of the rubber feedstock.

The goal of tyre pyrolysis is the complete conversion of all organic matter into TPO and non-condensable gas products, yielding an rCB free of pyrolysis residues. Process optimisation with this in mind has two major benefits:

1. By preventing the formation of pyrolysis residues, the surface of the recovered fillers is accessible in the elastomer phase, thus promoting reinforcing behaviour.
2. The biogenic carbon contained within the feedstock is recovered in the TPO and gas phases, where it adds value by reducing scope 1 emissions when using these materials directly, or after refinement as fuels.

rCB pyrolysis residues arise from two primary routes. Firstly, the incomplete removal of organic matter will leave oily residues on the surface of the rCB which, at high levels, have been conclusively linked to poor reinforcing properties [21]. Such residues pose little challenge to the industry, as they are easily identifiable by both the producer and end user using standard ASTM techniques, such as toluene discolouration (D1618) and thermogravimetric analysis (D8474) [22,23]. The second type of residue arises from polymer decomposition reactions that lead to the formation of additional fixed carbon, often referred to as carbonaceous residue [21,24,25]. Carbonaceous residue contents of >20 wt% have been reported and linked to a significantly reduced surface activity of rCB [8,26,27,28,29,30]. This residue is much harder to differentiate from the CB content of rCB, and so it often goes unnoticed. The net result of these pyrolysis residues is a reduction in ash content by the dilution effect, and this may be seen as an opportunity to meet the stringent ash content specifications applied in the rubber industry. The second aim of this study was to intentionally produce a high-carbonaceous-residue-content rCB from car tyres to explore an alternative strategy to meet ash specifications, and to assess the impact on morphological and in-rubber properties.

## 2. Materials and Methods

### 2.1. Tyre Granulate Preparation and Characterisation

Through careful selection of waste tyres, pyrolysis feedstocks were prepared from 100% car tyres or 100% truck tyres using Murfitts Industries commercial processing line at Lakenheath in the UK. The multi-stage process comprises shredding, granulation and separation steps to produce a ≤8 mm granulate with at least 99% steel and 98% fibre removal.

Proximate and ultimate analyses of twenty batches each of the car and truck feedstocks were completed. Proximate analysis followed the procedure outlined in ASTM D8474 [23], using a Perkin Elmer TGA4000 (Waltham, MA, USA) to yield volatile matter (VM), fixed carbon (FC) and ash contents.

### 2.2. rCB Materials Preparation

Pyrolysis trials were conducted using a two-stage continuous process comprising two in-sequence ETIA Technologies Spirajoule^®^ (Lacroix-Saint-Ouen, France) reactors with an overall throughput capacity of 90 kg/h. The operating conditions of the first stage were designed to complete ≥90% of the pyrolysis, with the second stage operating at a higher temperature to remove the residual organic matter from the raw rCB. Although the full process conditions cannot be disclosed for proprietary reasons, a brief description of how the process was optimised to produce the different rCB materials for this study is provided below.

Two grades of rCB were produced from the different feedstocks using process conditions optimised to minimise the rCB yield. This means that the target yield was based on the FC + ash content from the feedstock proximate analysis, producing material that is as free as possible from pyrolysis residues. The car tyre-derived rCB is referred to as Mi360+ and the truck tyre-derived grade as Mi360HP.

A further rCB material was produced from the car tyre feedstock but using very different process conditions to minimise the ash content. The different process conditions, including higher temperatures, were selected to promote the formation of carbonaceous residue on the rCB, thus diluting the inorganic components. This material is referred to as HCR (high carbonaceous residue).

All three materials were finalised in the same manner. The particle size was reduced to a target D97 of 10 ± 0.5 µm using a Hosokawa jet mill (Augsburg, Germany) before wet pelletising with a MARS Minerals (Mars, PA, USA) pin mixer and subsequent drying to produce pellets of <80 gf maximum hardness.

### 2.3. rCB Material Characterisation

A series of ASTM standardised techniques developed for carbon black and rCB were used to characterise the samples: this included BET surface area (D6556) [31], toluene discolouration (D1618) [22], bulk composition by TGA (D8474) [23], milled particle size distribution (WK87480) [32] and pellet hardness (D5230) [33].

To determine zinc and silicon contents, 250 mg of sample was digested on a hotplate with sulphuric and perchloric acids. When cold, they were transferred to plastic flasks where hydrofluoric acid was added to digest the silicon. Quantitative elemental analysis was performed using an Agilent 5800 (Agilent, CA, USA) inductively coupled plasma optical emission spectrometer (ICP-OES).

rCB morphology and inorganic distribution assessments were made using a Thermo Scientific F200i scanning (Waltham, MA, USA)/transmission electron microscope (S/TEM) fitted with a Bruker SDD XFlash 6.100 Energy Dispersive X-ray (EDX) detector (Berlin, Germany). The TEM was operated at 200 kV, and Velox software v3.19 used to acquire and process the elemental maps. Sample preparation involved a modified version of ASTM D3849 [34], as described by Grulke et al. [35].

### 2.4. In-Rubber Characterisation Protocol

The ASTM D3191 styrene butadiene rubber (SBR) formulation was used to evaluate the in-rubber behaviour of the rCB samples and an N550 reference [36]. A filler loading of 70 parts per hundred rubber (pphr) was used, as recommended in ASTM D8491 for rCB materials [37]. Compounds were produced using a 78 cc Haake Rheomix OS/3000 (Waltham, MA, USA) with Banbury style rotors set at 40 °C, with a rotor speed of 60 rpm.

Moving die rheometer (Alpha Technologies MDR2000, Akron, OH, USA) testing (to ASTM D5289) at 160 °C was used to assess the cure characteristics of each compound and to allow preparation of cured test sheets using a cure time to T90 + 5 min [38]. The cured rubber sheets were tested as follows:

- Tensile properties were determined using a Llyod LR5K (AMETEK, Bognor Regis, UK) following ASTM D412 [39].
- Shore A Hardness was determined using a Wallace H17A (Walace Instruments, Dorking, UK) in accordance with ASTM D2240 [40].
- Filler dispersion was assessed by examining surfaces cut with fresh razor blades at 250× magnification using a Hitachi TM3030 Scanning Electron Microscope (SEM) (Tokyo, Japan). Surface roughness plots and average surface roughness (Ra) values were generated using 3D-Image Viewer software v2.0 (Denshi Kougaku Kenkyusyo Co., Ltd., Tokyo, Japan).
- Strain sweeps were conducted using a Perkin-Elmer DM8000 (Waltham, MA, USA) configured in tension mode. During this step, 2 × 2 × 10 mm specimens were tested at 40 °C, 10 Hz and a double strain amplitude (DSA) range of ~0.04 to 4%.

## 3. Results and Discussion

### 3.1. Feedstock Characterisation

**Figure 1:**
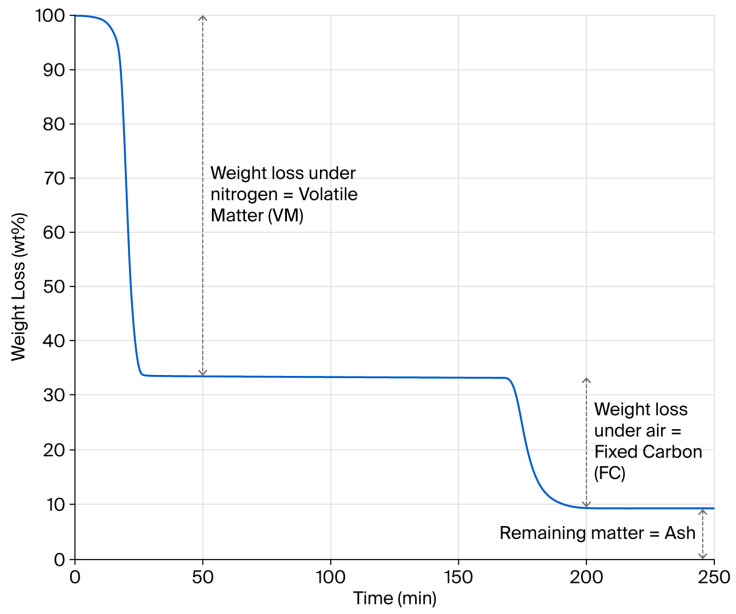
Example of rubber granulate TGA weight loss profile.

Estimates suggest that four million tons of waste tyres are generated globally every year [41]. Such statistics give the impression that this waste stream represents an almost infinite resource that can be misleading in relation to securing a consistent pyrolysis feedstock locally. Without doubt, compositionally consistent pyrolysis outputs necessitate a compositionally consistent feedstock. To emphasise this point, twenty batches of two compositionally different feedstocks, from 100% car or 100% truck tyres, were prepared, and their bulk compositional properties were determined through a proximate analysis. An example TGA weight loss profile is provided in Figure 1, showing the determination of volatile matter (VM), fixed carbon (FC) and ash contents, as adopted by Bowles and Fowler in their review of published rubber pyrolysis feedstocks [13]. Assuming pyrolysis is fully complete, the VM value derived from the TGA test should correlate with the hot gas yield of a pyrolysis process. Likewise, the sum of the FC and Ash values should offer a theoretical rCB yield, assuming that pyrolysis is complete and no process losses occur.

A summary of the bulk compositional properties of the two feedstocks is provided in Table 1, showing that the truck granulate contains a higher VM, higher FC and lower ash content. Two sample T-tests for each of the parameters yielded *p*-values of 0, confirming that the differences between the feedstocks are statistically significant. The narrow band of the 95% confidence intervals also demonstrates that it is possible to impart a high level of feedstock compositional control. Based on this data, the predicted pyrolysis rCB yields (FC + ash) are 35 wt% and 38 wt% when using truck and car granulate feedstocks, respectively. This data provides a valuable reference point when optimising the process to limit rCB contamination from pyrolysis residues. The data can also be used to predict the ash content of the resultant rCB. It is worthy of note that, even with the lower-ash-content truck feedstock, the theoretical ash content of the rCB is marginally above the industry standard of <20 wt%. This alone questions the practicality of the current specifications, especially when considering that the higher-ash-content car tyre rubber represents the bulk of the waste stream.

### 3.2. rCB Characterisation

The properties of the three different rCB materials are summarised in Table 2. The rCB yields of samples optimised for low residue content, Mi360HP (truck) and Mi360+ (car), were in good agreement with the theoretical yields derived from the proximate analysis of the feedstock. Compared to the Mi360+, the high carbonaceous residue (HCR) sample produced from the same feedstock had an elevated FC content, confirming that the changes in the pyrolysis process conditions had the intended effect of increasing the carbonaceous residue content. The difference in yield between the Mi360+ and HCR suggests that approximately 17 wt% of the feedstock was converted into carbonaceous residue for the latter. All samples had comparably low organic residue contents, as demonstrated by both the toluene extract transmittance and VM content values. This parameter can therefore be disregarded when considering the differences in rubber reinforcing behaviour between the samples. The VM fraction will contain a contribution from moisture and a small amount of residual organic matter.

The measured ash contents of the Mi360 grades were slightly below that predicted from the feedstock analysis. However, a good agreement between theoretical and measured rCB yields would suggest that the residue content is very low. The discrepancy may arise from natural variability (i.e., only one sample measured compared to the average of twenty feedstock samples) and may also result from the different temperature profiles used for the TGA test and pyrolysis process. The key point is that the deposition of carbonaceous residues within the car tyre-derived HCR sample reduced the ash content in line with the truck tyre-derived Mi360HP. This provides the basis for assessing the relative effects of ash and carbonaceous residues on the reinforcing behaviour of the rCB materials.

**Figure 2:**
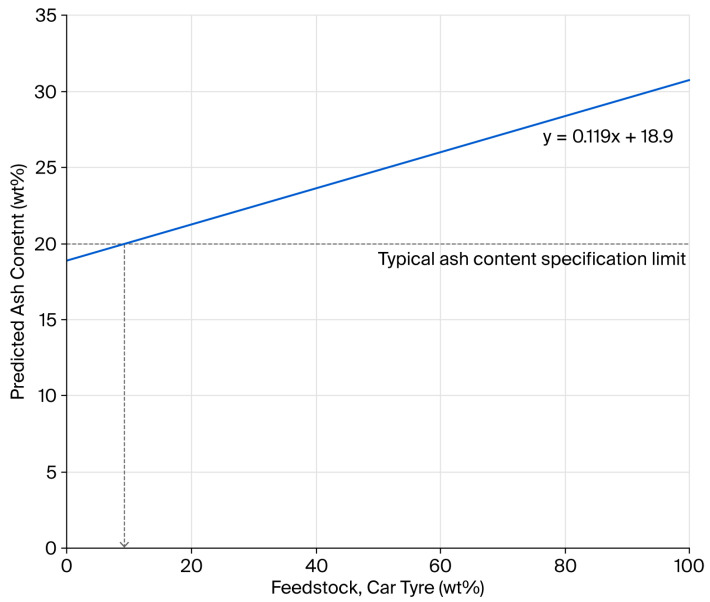
Relationship between rCB ash content and the percentage of car tyres used in a feedstock blend with truck tyres.

One of the strategies adopted by rCB producers to meet ash content targets is to dilute the higher-ash-content car tyre feedstock with lower-ash-content truck tyres. Using the ash contents of the low-residue Mi360HP and Mi360+, predictions can be made on the necessary blend ratio to meet the <20 wt% ash content, as seen in Figure 2. The prediction assumes that, by blending the feedstock, the residue content of the rCB does not differ to those produced from the segregated waste streams. Based on this data, a feedstock blend containing no less than 91 wt% truck tyres would be necessary to meet the target and is by no means reflective of the relative abundance of this waste tyre category. Such ash content targets almost necessitate the need to form carbonaceous residue during pyrolysis.

Of course, ash is a poor descriptor of the inorganic components contained within an rCB product. Most of the inorganic matter arises from the use of silica filler and zinc oxide additives [8,13,16,17,18,21], as confirmed by an ICP-OES analysis of acid digests. Car tyres contain a much higher loading of silica, but it is also apparent that silica has found use in truck tyres. The data also confirm that truck tyres contain a higher zinc loading compared to car tyres. The rCB materials will also contain low levels of other inorganic matter, such as iron and other trace metals originating from the feedstock, but these were not quantified as part of this study.

**Figure 3:**
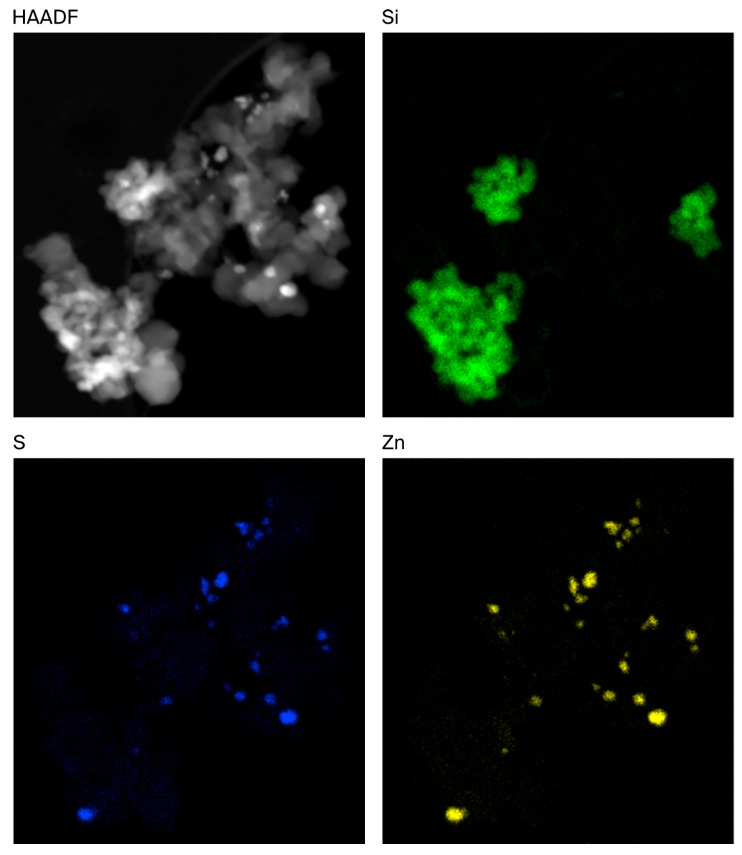
STEM HAADF (electron) image of rCB fused agglomerate alongside silicon, sulphur and zinc distribution maps.

STEM/EDX mapping of the rCB samples was performed to better understand the distribution of these inorganic components. An example of an Mi360+ fused agglomerate is displayed in Figure 3, showing that the silica clusters are intimately entwined amongst the CB aggregates. Zinc was found to be in the form of zinc sulphide, appearing as discrete particles at the surface of the fillers, in agreement with other microscopy assessments of rCB [16,18]. As such, no evidence was identified to suggest that the zinc sulphide particles influenced agglomeration. The pyrolysis gas phase contains an abundance of hydrogen sulphide, with levels of up to 5.1 vol% reported [42,43]. It is highly likely that the simple reaction between zinc oxide and hydrogen sulphide yields the observed particles of zinc sulphide:

ZnO + H_2_S → ZnS + H_2_O

The conversion of ZnO to ZnS may not be true for all pyrolysis processes, depending on the process conditions utilised.

The same observations were made for the Mi360HP, but with lower amounts of silica present.

**Figure 4:**
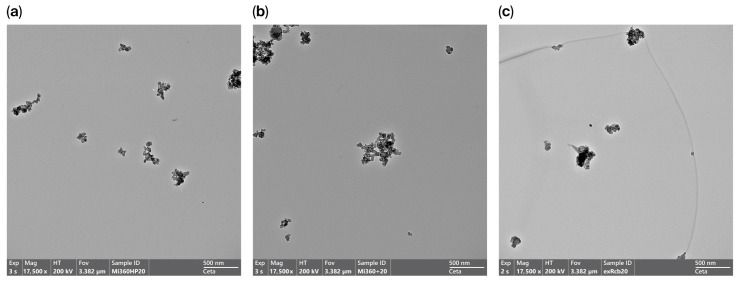
TEM images showing rCB morphology; (**a**) Mi360HP, (**b**) Mi360+ and (**c**) HCR.

The presence of carbonaceous residue within the HCR sample had a dramatic effect on morphology, with the aggregate nature much less apparent in many of the particles studied, as seen in Figure 4. This observation aligns with the BET measurements of the raw rCBs (prior to milling and pelletising), with the HCR exhibiting 46% less surface area than the Mi360+ produced from the equivalent feedstock. As structure level and surface area are regarded as fundamental properties of rubber reinforcement [44,45,46], the HCR would be expected to exhibit an inferior reinforcing behaviour.

The milled particle size target of d97 10 ± 0.5 µm was achieved for all samples. This parameter is directly related to dispersion [47], and so it is important that all were size-reduced to the same level to allow for a fair comparison of in-rubber properties. All samples were noted to exhibit an increased surface area post milling: Mi360HP by 13%, Mi360+ by 4% and HCR by 68%. These data suggest that the presence of carbonaceous residue is linked to a significant increase in milled surface area, closing the gap between the HCR and the Mi360 grades. Traditional carbon black colloidal tests have long been established as poor predictors of the in-rubber properties of rCB [8,21]. These observations further challenge the value of surface area in rCB specifications, especially when considering that the end-user cannot test the material in the pre-milled state.

Finally, all samples were successfully pelletised to industry standard levels to facilitate proper handling and rubber compounding behaviour.

### 3.3. In-Rubber Characterisation

The rCB materials were compounded into the SBR-based ASTM D3191 formulation to investigate the effects of the compositional differences on their in-rubber properties, as seen in Table 3. Comparing the properties of Mi360HP and Mi360+ offers insight into the effect of ash content, whilst the differences between the Mi360+ and HCR are wholly related to the carbonaceous residue content.

**Figure 5:**
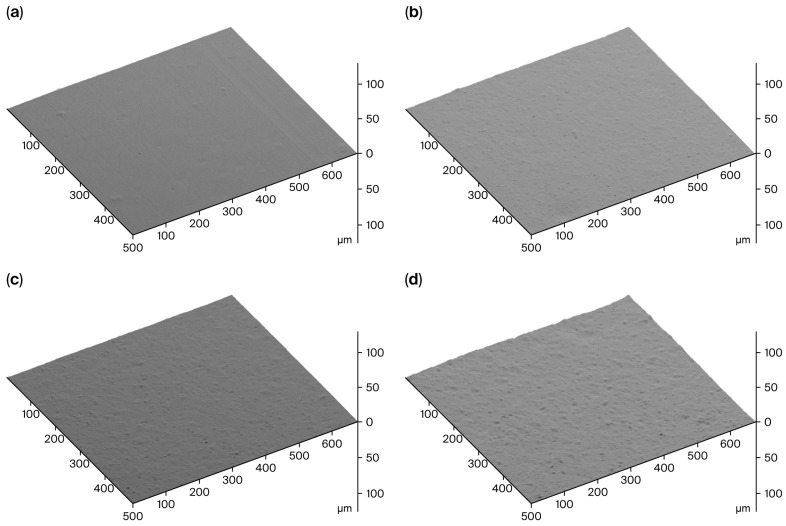
SEM Surface roughness maps of sectioned surfaces of model SBR compounds containing (**a**) N550, (**b**) Mi360HP, (**c**) Mi360+ and (**d**) HCR.

Surface roughness maps indicating the level of filler dispersion are shown in Figure 5. The dispersion of the Mi360 grades almost matched that of the N550. It should be noted that N550 is one of the easiest grades to disperse, with its low surface area and high structure level. Although milled to the same particle size, the HCR was found to be harder to disperse compared to the Mi360 grades. This would suggest that the Mi360 grades underwent a level of agglomerate breakdown during the mixing process. It is likely that the presence of carbonaceous residue on the HCR prevents effective de-agglomeration, leading to an inferior dispersion. The presence of larger agglomerates has been linked to poorer mechanical properties [14], as discussed below.

The mechanical properties of the Mi360HP were a good match to the N550 reference. The higher ash content Mi360+ had significantly reduced stiffness values. Most of the difference between the Mi360HP and the Mi360+ is attributable to the higher silica content of the latter. Silica, without a coupling agent, is known to be less reinforcing than carbon black [19]. In addition, silica can remove soluble zinc from its normal accelerator activating function, resulting in a reduced crosslink density [18]. Another factor is likely to be the different grades of CB recovered from the truck and car tyre feedstocks. Truck tyres utilise more N100 series in the tread sections and N300 series in the sidewalls to provide the necessary abrasion and stiffness characteristics. Car tyres tend to use N300 series in the treads and N500 to N600 in the sidewalls. In short, the CB content of Mi360HP is expected to be more reinforcing than the CB content of Mi360+.

**Figure 6:**
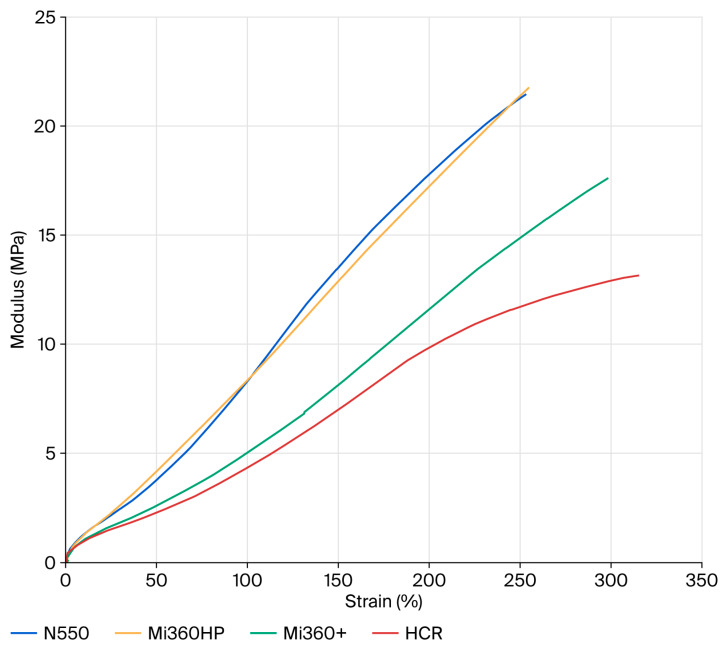
Tensile curves of the different rCB variants compared to N550 in the model SBR formulation.

Although the ash content matched that of the Mi360HP, the deposition of carbonaceous residues in the HCR reduced the hardness and modulus values further compared to the Mi360+. This conclusively demonstrates that carbonaceous residues are more detrimental to reinforcing behaviour than ash. In particular, there is an apparent loss of filler–polymer interaction at higher tensile strains above approximately 200%, as seen in Figure 6. This aligns with the loss of structure identified during the TEM assessment and reports that the carbonaceous residue masks the surface activity of the original carbon black.

**Figure 7:**
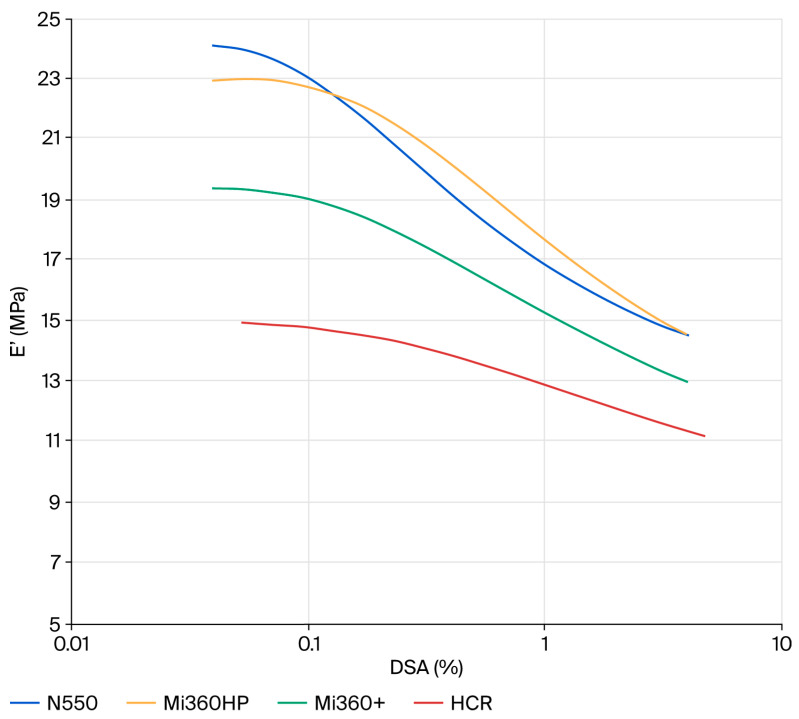
Strain dependency plots of the different rCB variants compared to N550 in the model SBR formulation.

Given that the ultimate goal of tyre pyrolysis is to provide raw materials back into the tyre industry, it was prudent to evaluate the dynamic properties of the rCB materials. Elastic modulus plots showing the non-linearity of compounds filled with N550 and different rCB materials are provided in Figure 7. At a low strain (E′_0_), the dynamic stiffness contains the full contribution of the filler network. As the dynamic strain increases, the filler–filler interactions are gradually disrupted, leading to a plateau at E′_∞_. This phenomenon is known as the Payne effect [48]. The difference between the low and high strain modulus (∆E′ = E′_0_ − E′_∞_) can therefore be used as a measure of the filler network. Assuming equal filler loadings and dispersion, the two key parameters that influence the level of filler–filler interaction are the surface area and surface chemistry. For standard grades of CB, ∆E′ scales directly with surface area, as all exhibit the same surface chemistry. Given the higher surface area of the rCB materials, they would be expected to exhibit higher ∆E′ values than N550 if their surface chemistry was the same. This is clearly not the case for the rCBs, with all exhibiting a lower filler networking efficiency, in line with previous studies that attributed the networking reduction to the presence of carbonaceous residue masking the active sites of CB [8,21]. Considering the effect of ash, there is a general trend for reducing filler–filler interactions from N550→Mi360HP→Mi360+ as ash content increases. In some respects, the rCBs behave like a dual-filler system, with silica and zinc sulphide particles acting to disrupt the CB network. The clear effect of carbonaceous residue is seen when comparing the HCR to the Mi360+, with further reduced filler–filler interactions for the former. This will be a function of both the reduced surface area and surface activity of the HCR.

At a high strain (E′_∞_), the dynamic stiffness is a combination of gum modulus, hydrodynamic effects and filler–polymer interactions [48]. Considering the formulation and filler loadings were constant, E′_∞_ can be used as a measure of the filler–polymer interaction. In this case, the Mi360HP exhibits a similar level of filler–polymer interaction as the N550, in good agreement with the physical properties data. As the silica level increases for the Mi360+, the filler–polymer interaction lessens. A previous study has shown that the silica content of an rCB is amenable to silane coupling, offering a route to enhancing the polymer–filler interaction of residue-free rCB [49]. As for the other parameters, the effects of carbonaceous residue on reducing the polymer–filler interaction were pronounced.

## 4. Conclusions

This study demonstrates that it is possible to impart a high degree of control to prepare statistically different feedstocks to produce different grades of rCB. Furthermore, a detailed understanding of the feedstock composition can be used to predict both rCB yields and rCB ash contents, offering useful tools in the optimisation of a pyrolysis process. The fixed carbon and ash content of a pyrolysis feedstock should correspond to the rCB yield, if free from organic and carbonaceous residues. This approach was used in the production of two grades of rCB, Mi360HP and Mi360+, produced from truck and car tyres, respectively. The primary difference between the two is their ash content, at 19% for the Mi360HP and 31% for the Mi360+. The term ash is a poor descriptor of the inorganic components of rCB, with TEM/EDX and ICP-OES analyses confirming the bulk to be silica filler clusters entangled with the CB aggregates, being more prevalent in the car tyre derived Mi360+. The other key inorganic component was zinc sulphide, found to reside as discrete particulates on the surface of the filler aggregates.

It has long been established that, for equivalent colloidal properties, rCB does not exhibit the same in-rubber behaviour as CB. Clearly, ash content, and to be more precise silica content, is a contributing factor. Silica, without a coupling agent, exhibits a lower polymer interaction compared with CB, manifesting as lower quai-static and dynamic modulus values for the Mi360+ when compared to the lower-silica-content Mi306HP. Ash content, therefore, performs an important role in the development of rCB specifications. However, the typical rCB ash content specifications of <20 wt% are unrealistic from UK waste tyres, especially when considering that the theoretical rCB ash contents from truck and car tyres were determined as 20 and 35 wt%, respectively. Furthermore, the Mi360HP was close to this limit (19 wt%), but still closely matched the tensile properties and filler–polymer interaction of the N550.

To demonstrate that ash is far from the only consideration regarding rCB reinforcing behaviour, a high carbonaceous residue (HCR) sample was prepared from car tyres to dilute the ash content to 19 wt%, i.e., within typical ash content specifications. This material ranked worse than the high-silica-content Mi360+ for all of the in-rubber properties, including dispersion, tensile and dynamic behaviour. TEM and colloidal assessments identified the carbonaceous residues as significantly reducing the surface area and structure of the rCB, both of which are fundamental to rubber reinforcement. Although ash content attracts a lot of attention, this study conclusively shows that carbonaceous residues are more detrimental to rCB performance.

Many have studied rCB ash reduction [16,17,50,51,52], but little attention has been given to controlling and limiting carbonaceous residues. This is likely linked to the availability of well-established test methods for the easy determination of ash content [23,53]; however, the same is not true for carbonaceous residues, which are difficult to differentiate from the CB content of an rCB. A fast and reliable method for carbonaceous residue determination would be extremely beneficial to the industry, offering the missing piece of the jigsaw in the classification and specification debate. Furthermore, the ability to silane-couple the silica component of a residue-free rCB provides an opportunity to benefit from the growing silica content of road tyres.

## Figures and Tables

**Table 1:** Mean proximate analysis values with 95% confidence intervals in brackets.

Parameter	Unit	Truck Granulate	Car Granulate
VM Content	wt%	64.7 (64.0→65.3)	62.5 (61.8→63.0)
FC Content	wt%	28.2 (27.6→28.8)	24.5 (23.4→25.7)
Ash Content	wt%	7.1 (6.5→7.8)	13.1 (12.1→14.0)
Theoretical rCB Yield (FC + Ash)	wt%	35.3	37.6
Theoretical rCB Ash Content	wt%	20.1	34.8

**Table 2:** Summary of rCB sample properties.

Parameter	Unit	Truck rCB (Mi360HP)	Car rCB (Mi360+)	Car rCB HCR
Measured rCB Yield	wt%	36	38	55
Toluene Transmission	%	98	99	100
VM–TGA	wt%	1.3	1.7	1.8
FC–TGA	wt%	79.0	67.6	79.4
Ash–TGA	wt%	18.9	30.8	18.7
Si–ICP	wt%	3.7	9.1	7.0
SiO_2_–Calculated *	wt%	7.9	19.5	15.0
Zn–ICP	wt%	5.0	3.5	2.5
ZnS–Calculated *	wt%	7.5	5.2	3.7
BET Surface Area–Raw rCB	m^2^/g	79.3	82.7	44.7
BET Surface Area–rCB	m^2^/g	89.9	85.8	75.0
Milled Particle Size, d97	µm	10.3	9.5	10.3
Average Pellet Hardness	gF	33	35	39

* ICP derived atomic mass data converted to molecular mass of associated chemical.

**Table 3:** Summary of in-rubber properties.

Parameter	Unit	N550	Truck rCB (Mi360HP)	Car rCB (Mi360+)	Car rCB HCR
Dispersion–Ra	µm	0.34	0.43	0.46	0.75
Shore A Hardness	°	80	78	76	70
M100%	MPa	8.38	8.51	5.08	4.74
M200%	MPa	17.92	17.39	11.72	10.72
Break Stress	MPa	21.6	21.3	17.1	13.6
Break Strain	%	252	247	296	303
E′_0_	MPa	24.1	23.0	19.4	15.0
E′_∞_	MPa	14.6	14.6	13.0	11.3
∆E′	MPa	9.5	8.4	6.4	3.8
Tan δ_max_	-	0.19	0.18	0.17	0.16

## Data Availability

The original contributions presented in this study are included in the article. Further inquiries can be directed to the corresponding author.

## Abbreviations

The following abbreviations are used in this manuscript:

rCB	Recovered carbon black
TPO	Tyre pyrolysis oil
CB	Carbon black
VM	Volatile matter
FC	Fixed carbon
HCR	High carbonaceous residue
BET	Brunauer–Emmett–Teller
TGA	Thermogravimetric analysis
ICP-OES	Inductively coupled plasma optical emission spectrometer
EDX	Energy dispersive X-ray
S/TEM	Scanning/transmission electron microscope
MDR	Moving die rheometer
SEM	Scanning Electron Microscope
